# Design of a Tunable Ultra-Broadband Terahertz Absorber Based on Multiple Layers of Graphene Ribbons

**DOI:** 10.1186/s11671-018-2552-z

**Published:** 2018-05-09

**Authors:** Zenghui Xu, Dong Wu, Yumin Liu, Chang Liu, Zhongyuan Yu, Li Yu, Han Ye

**Affiliations:** 1 State Key Laboratory of Information Photonics and Optical Communications, Beijing University of Post and Telecommunications, Beijing, 100876 China; 2 School of Science, Beijing University of Post and Telecommunications, Beijing, 100876 China

**Keywords:** Thz absorption, Surface plasmons, Metamaterials, Tunable Thz devices

## Abstract

We propose and numerically demonstrate an ultra-broadband graphene-based metamaterial absorber, which consists of multi-layer graphene/dielectric on the SiO_2_ layer supported by a metal substrate. The simulated result shows that the proposed absorber can achieve a near-perfect absorption above 90% with a bandwidth of 4.8 Thz. Owing to the flexible tunability of graphene sheet, the state of the absorber can be switched from on (absorption > 90%) to off (reflection > 90%) in the frequencies range of 3–7.8 Thz by controlling the Fermi energy of graphene. Moreover, the absorber is insensitive to the incident angles. The broadband absorption can be maintained over 90% up to 50°. Importantly, the design is scalable to develop broader tunable terahertz absorbers by adding more graphene layers which may have wide applications in imaging, sensors, photodetectors, and modulators.

## Background

In recent years, the terahertz band has become one of the most interesting platform because of the huge application in spectroscopy, medical imaging, modulators, security, and communication [1–3]. The terahertz absorber is an important branch, which can find practical applications in the above fields [4–6]. However, the narrow bandwidth, low absorption efficiency, and non-adjustable absorption performance of the absorbers limit their applications in practice greatly. In order to better expand the application of terahertz absorber, more new devices and materials are urgently required. Graphene, as a two-dimensional material with the honeycomb lattice structure, has become one of the most promising materials due to its tunability of conductivity controlled by electric field, magnetic field, gate voltage, and chemical doping [7–14]. Especially, graphene can support surface plasmons in the terahertz ranges. Compared with the traditional surface plasmon material, graphene surface plasmons have the advantage of low losses, flexible tunability and so on [15–19].

Owing to the superiority of graphene materials in terahertz absorbers, there are some graphene absorbers that have been proposed and demonstrated [20–34]. Theoretical analysis confirms that a single layer of graphene is optically transparent and it has an absorption of 2.3% [35–37]. To enhance the confinement of the electromagnetic energy, periodical patterned graphene structures have been designed such as net-shaped [20–22], anti-dots [23], and cross-shaped [32]. However, these absorbers are deeply dependent on complex structured graphene which result in fabrication difficulty. Moreover, the band available for operation is very narrow, and most of the works reported do not have bandwidth of more than 1.5 Thz [20–28]. In order to broaden the bandwidth, several multilayer graphene structures have been proposed. However, the reported multilayer structures are also dependent on very complex structure of the graphene, and the operation bandwidths are not long enough [32–34]. In addition, Zhao et al. designed a switchable terahertz absorber for the application of amplitude modulator [25]. By controlling the chemical potential of graphene from 0 to 0.3 eV, the state of the designed structure can be switched from absorption (> 90%) to reflection (> 82%) in the frequencies range of 0.53–1.05 Thz. But the switching intensity is not high enough, and the modulation bandwidth is very narrow, which limit its further application in practice.

In this paper, we present a tunable graphene-based terahertz absorber composed of multilayer graphene which can achieve an ultra-broadband absorption over 90% in the frequencies range of 3–7.8 Thz. The average absorptivity of the absorber is higher than 96.7%. Besides, the proposed absorber has higher switching intensity, the absorption amplitude can be tuned from near-perfect absorption (> 90%) to high reflection (> 90%) by changing the Fermi energy of graphene layer in the whole bandwidth of 4.8 Thz. When the Fermi energy of graphene is 0 eV, the proposed structure will be a near-perfect reflector with a reflection more than 97% in the high frequency band (about 5.5 Thz later). In addition, the absorber is independent to the incident angles with the absorption more than 90% up to 50°. To our best knowledge, we first propose the two-dimensional multi-layer graphene/dielectric structure to realize an ultra-broadband absorption. The proposed absorber is simple which do not depend on complex patterned graphene, and the design provides great convenience for the fabrication of multilayer graphene structures [38, 39]. Importantly, the design is scalable to develop broader tunable terahertz absorbers by adding more graphene layers, which may have wide application in terahertz optoelectronic devices.

## Methods

**Fig. 1:**
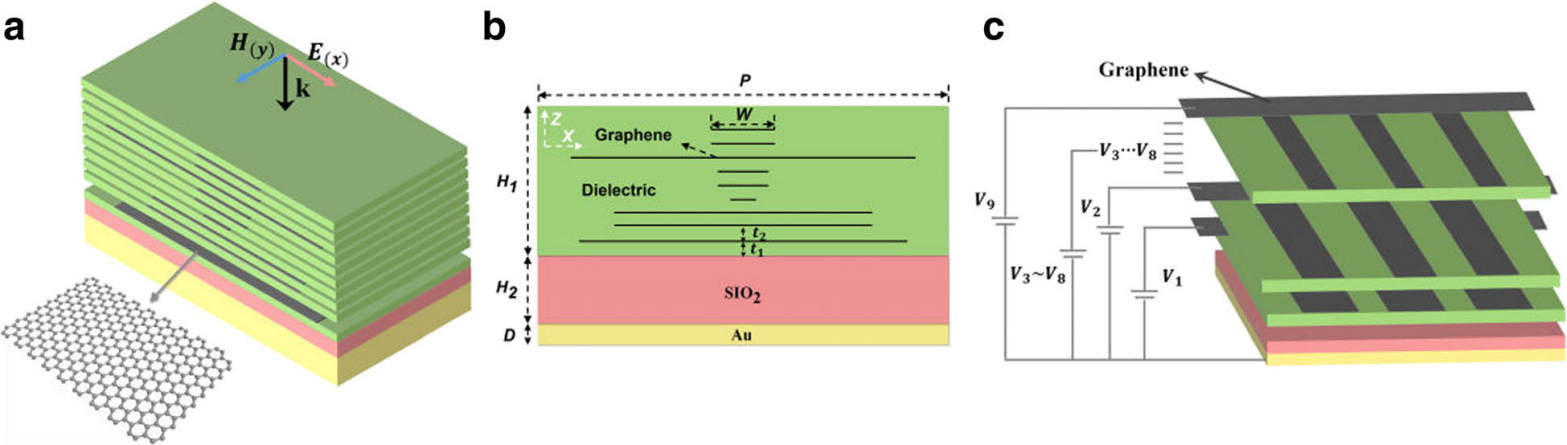
**a** Schematic diagram of the graphene-based broadband absorber. **b** Cross section of the absorber with the parameters used for calculation. **c** The schematic of the external bias circuit. The branches of the voltage (*V*_1_~*V*_9_) are connected to diferent graphene layers, respectively

The diagram of the proposed structure is shown in Fig. 1, which consists of multi-layer graphene embedded into the dielectric on the SiO_2_ layer and a thick metallic reflecting plate on the bottom. As shown in Fig. 1, on the top, graphene with different width (*W*) is embedded in the dielectric at a certain gap *t*_2_ (*t*_2_ = 2 μm). The width *W* of each graphene is 5, 5, 27, 4, 4, 2, 21, 21, and 26 μm, respectively (from top to bottom). Each layer is symmetrical about the *z*-axis. The distance *t*_1_ between the bottom of the graphene layer and the SiO_2_ layer is 2 μm. The thickness of the dielectric is *H*_1_. The middle layer is SiO_2_ with a thickness of *H*_2_. The bottom is a metallic film with a thickness of *D*. The period of the unit is *P.* These initial values of the structure parameters are set to *H*_1_ = 21 μm, *H*_2_ = 7 μm, *D* = 0.5 μm, *P* = 32 μm. The bottom metallic material is gold, and its permittivity can be properly represented by the Drude model in terahertz range as follows:

1 $$ \varepsilon ={\varepsilon}_{\infty }-\frac{\omega_p^2}{\omega^2+ i\omega \gamma} $$

where the value of constant permittivity *ε*_∞_, plasma frequency *ω*_*p*_, and collision frequency *γ* are set to 1, 1.38 × 10^16^ rad/s, and 1.23 × 10^13^ s^− 1^, respectively. The permittivity of the dielectric material and SiO_2_ material are set to 3 and 4, respectively.

In the simulation, graphene is treated as an ultra-thin film embedded in the dielectric. The complex graphene surface conductivity dominated by interband and intraband contributions can be calculated by using the Kubo formula [40]:

2 $$ {\displaystyle \begin{array}{l}\sigma \left(w,{E}_f,\tau, T\right)={\sigma}_{\mathrm{inter}}+{\sigma}_{\mathrm{intra}}=\frac{je^2\left(w-j{\tau}^{-1}\right)}{\pi {\mathrm{\hslash}}^2}\times \\ {}\left[\frac{1}{{\left(w-j{\tau}^{-1}\right)}^2}\underset{0}{\overset{\infty }{\int }}\frac{\partial {f}_d\left(\varepsilon \right)}{\partial \varepsilon }-\frac{\partial {f}_d\left(-\varepsilon \right)}{\partial \varepsilon } d\varepsilon -\underset{0}{\overset{\infty }{\int }}\frac{f_d\left(-\varepsilon \right)-{f}_d\left(\varepsilon \right)}{{\left(w-j{\tau}^{-1}\right)}^2-4{\left(\varepsilon /\mathrm{\hslash}\right)}^2} d\varepsilon \right]\\ {}\kern0em \end{array}} $$

where ${f}_d\left(\varepsilon \right)={\left({e}^{\left(\varepsilon -{E}_f\right)/{k}_BT}+1\right)}^{-1}$ is the Fermi-Dirac distribution, *w* is the radian frequency, *ε* is the energy, *k*_*B*_ is the Boltzmann’s constant, *τ* is the carrier relaxation time, *T* is the temperature (*T* = 300 K in our paper), ℏ is the reduced Plank’s constant, and *E*_*f*_ is Fermi energy. The Kubo formula (2) indicates that the complex graphene surface conductivity can be adjusted by Fermi energy *E*_*f*_. The graphene Fermi energy of each layer can be individually controlled by the biased voltage, the relation between *E*_*f*_ and biased voltage can be written as [41, 42]:

3 $$ \left|{E}_f\left({V}_n\right)\right|=\mathrm{\hslash}{v}_F\sqrt{\pi \left|{a}_0\left({V}_n-{V}_0\right)\right|}\kern1.5em \left(n=1,2,3..,9\right) $$

where *v*_*F*_ = 0.9 × 10^6^m/s is the Femi velocity, *V*_0_ is the voltage offset [41], ${a}_0=\frac{\varepsilon_0{\varepsilon}_d}{ed}$, *a*_0_ is the capacitive model of the structure, where *ε*_0_ is the permittivity in vacuum. *ε*_*d*_ is the permittivity of dielectric, *d* is the height of dielectric, and *e* is the charge of an electron. *V*_*n*_(*V*_1_~*V*_9_), that is, the voltage applied to the graphene can be obtained from the additional circuit of Fig. 1c. According to Formula (2) and (3), graphene’s surface conductivity can be controlled by the applied voltage. Then, based on the Ampere’s law in stationary regime and Ohm’s law, the permittivity of graphene can be obtained as [43]:

4 $$ {\varepsilon}_g=1+i\frac{\sigma_g}{t_g{\varepsilon}_0\omega } $$

In which *t*_*g*_ is the thickness of the graphene, *ε*_0_ is the permittivity of vacuum, and *σ*_*g*_ is the surface conductivity of the graphene. According to Formula (4), the permittivity of graphene can be obtained by the surface conductivity, which can also be obtained by the applied voltage. Therefore, Formula (2–4) indicate that the electromagnetic properties of graphene can be dynamically controlled by the applied voltage, leading to that the absorption characteristics of the structure can also be dynamically controlled.

To investigate the absorption performance of the designed structure, we implement the numerical simulations using two-dimensional FDTD. In our simulation, we set the structure to a periodic boundary condition in the x direction. A beam of terahertz plane wave is incident to the model normally along the *z* direction with its electric field E along *x* direction. The Bloch boundary condition is applied to the oblique incidence in the periodic structure. We use 1-R-T to calculate the absorption of the model, where R and T represent reflectivity and transmissivity, respectively. Since the thickness of the metal is much greater than the skin depth of incident light in the metal, the transmissivity T is zero. Thus, we simplify the calculation formula for 1-R.

## Results and Discussion

**Fig. 2:**
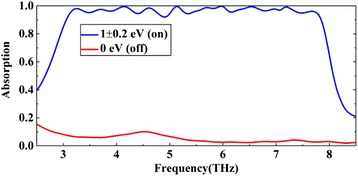
The calculated absorption spectra of the proposed absorber, where the blue line represents the absorption with high voltage and the red line indicates the absorption with no voltage applied

Firstly, we tune the voltage of each graphene layer to achieve perfect absorption (from top to bottom, we fine adjust the Fermi energy *E*_*f*_ of the each graphene layer to 0.9, 0.9, 1.1, 0.8, 0.8, 1.1, 1.1, 0.9, and 0.8 eV). As shown in Fig. 2, from 3 to 7.8 Thz, the proposed structure has a broadband absorption above 90% within a bandwidth of 4.8 Thz. The FWHM of the absorber is 5.4 Thz. The bandwidth is about $\frac{BW}{f_0}\times 100\%$ = 88.8% of central frequency (here, *BW* is the bandwidth and *f*_0_ is central frequency). We also calculate the average absorptivity of the absorber, which is as high as 96.7%. On the other hand, with the *E*_*f*_ = 0 eV, the proposed structure will be a near-ideal reflector with reflection more than 90% over the whole operation bandwidth, and in the high frequency band (about 5.5 Thz later), the reflection even more than 97%. Of course, we can also tune the voltage of each graphene layer to get the desired amplitude which may have potential applications in some areas.

**Fig. 3:**
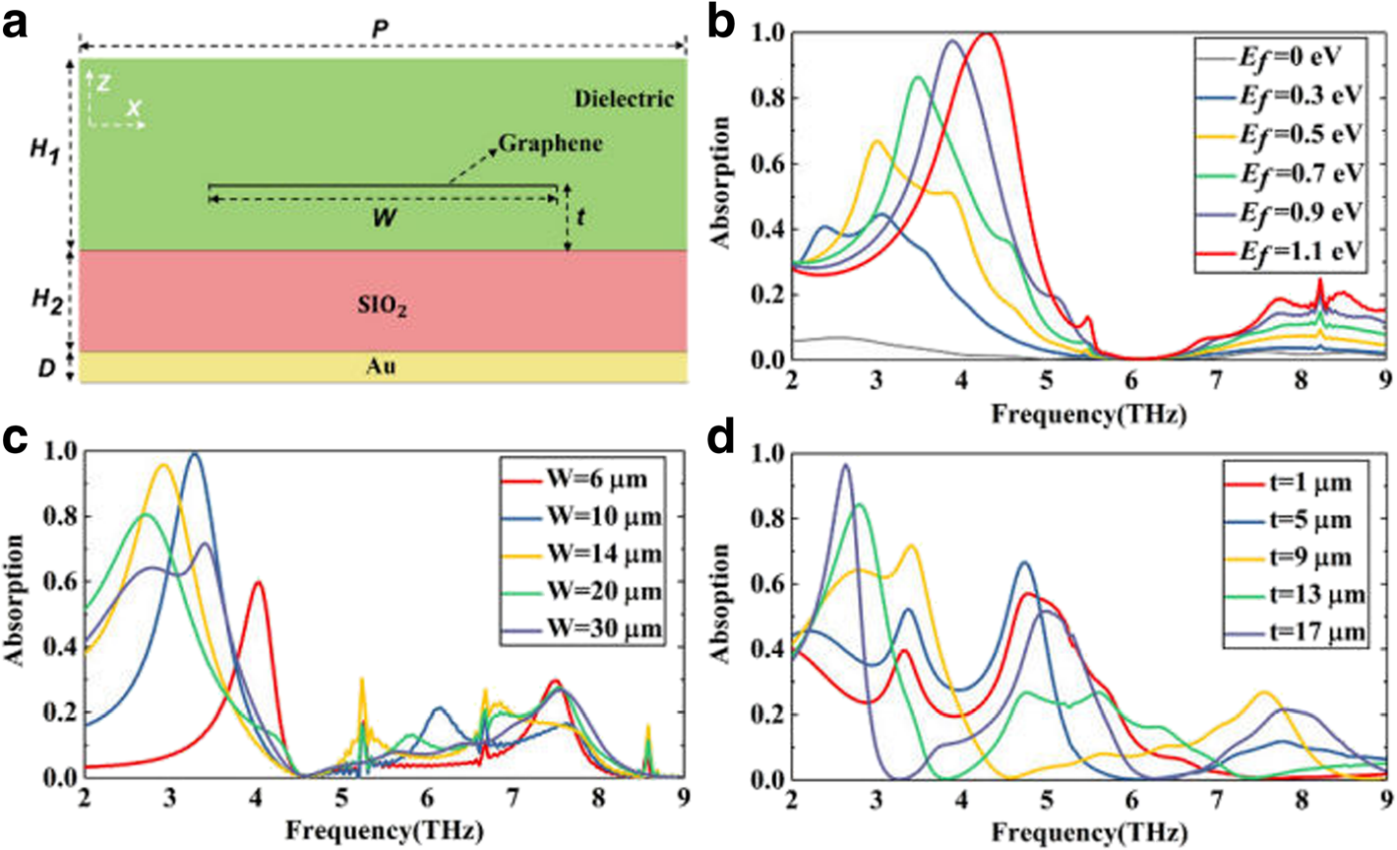
**a** Schematic diagram of a single-layer graphene structure. **b**–**d** The absorption of the structure width different Fermi energy *E*_*f*_, width *W*, and position *t* of the graphene sheet, respectively

In order to explain the near-perfect absorption in an ultra-broad bandwidth, firstly, we discuss the situation of a single graphene layer. As shown in Fig. 3a, we design the structure with only one single graphene layer embedded into the dielectric. Based on graphene surface plasmons, we investigate the effect of graphene-related parameters on the absorption performance of absorber, including the Fermi energy *E*_*f*_, the width *W*, and the position *t* of graphene.

Figure 3b shows the influence of graphene Fermi energy *E*_*f*_ on the absorption spectrum with fixed *W* and *t*. As the increase of *E*_*f*_, the graphene surface plasmon resonance becomes stronger, the absorption of the structure is higher correspondingly. The absorption peak even more than 99% at 4.3 Thz with the *E*_*f*_ = 1.1 eV. And the resonance absorption peak moves to a higher frequency, blue shift. Similarly, Fig. 3c, d shows the absorption spectrum of the structure with different *W* or *t* with unchanged *E*_*f*_ . By varying the *W* or *t* of the graphene layer, the amplitude and frequency of the resonance peak are changed respectively. This phenomenon can be explained by the circuit theory [28]. In this theory, graphene is described as a shunt admittance, then the equivalent circuit of the structure can be modeled with transmission lines and graphene admittance. According to previous work [28], the graphene admittance can be changed by the width *W* and the Fermi energy *E*_*f*_ of the graphene. In addition, the admittance of the transmission lines corresponding to dielectric are related to the thickness of the dielectric. In our structure, the dielectric is separated by graphene layer. Thus, the position *t* of the graphene layer also affects the input admittance of the structure.

As we discussed above, due to the influence of graphene-related parameters on the input admittance of the structure, the resonance absorption peaks of the model are also influenced. If the input admittance of the structure matches to the free-space admittance, the near-perfect absorption at a certain frequency is achieved.

**Fig. 4:**
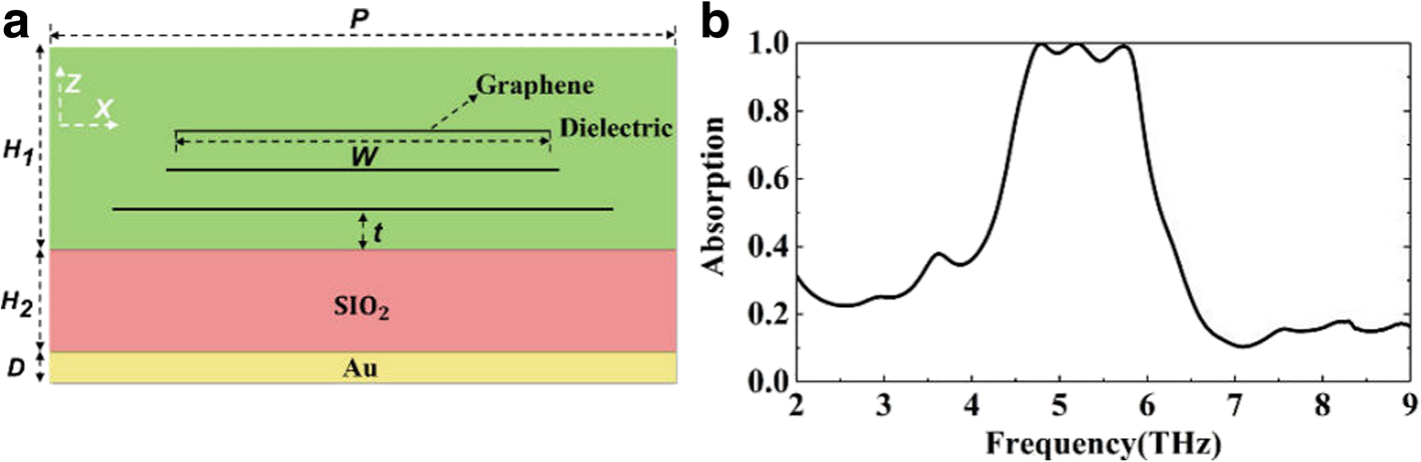
**a** Schematic diagram of a three-layer graphene structure. **b** The calculated absorption spectra of three-layer graphene structure

Then, in order to achieve broadband absorption, we need to allow the resonance absorption peaks which achieve the admittance matching close to each other. As the absorption peaks are close enough to merge, a broadband absorption is obtained. Therefore, we add graphene layers to get more resonance absorption peaks. And at the same time, we adjust the parameters that affect the resonance peak, including *E*_*f*_, *W*, and *t* to implement admittance matching. We first add two layers of graphene. As shown in Fig. 4a, three layers of graphene with different width *W* are embedded in the dielectric. There is a certain interval *t* between different layers of graphene or the bottom graphene from the dielectric. We adjust the graphene-related parameters to the appropriate values, where we set *t* = 2 μm, *E*_*f*_ = 0.9 eV, and *W* = 26, 21, and 20 μm, respectively (from bottom to top).

**Fig. 5:**
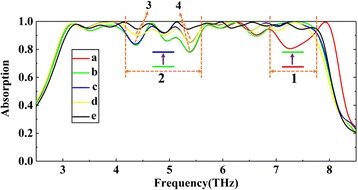
(a)–(e) show the gradual adjustment process for perfect absorption. Fermi energy of each layer of graphene (from bottom to top) are set as (a) [1] eV, (b) [1, 1, 1, 0.8, 1, 1, 1, 1, 1] eV, (c) [1, 1, 1, 0.8, 1, 1, 1, 0.9, 0.8] eV, (d) [1, 1, 1, 0.8, 0.8, 1, 1, 0.9, 0.8] eV, and (e) [0.9, 0.9, 1.1, 0.8, 0.8, 1.1, 1.1, 0.9, 0.8] eV

**Fig. 6:**
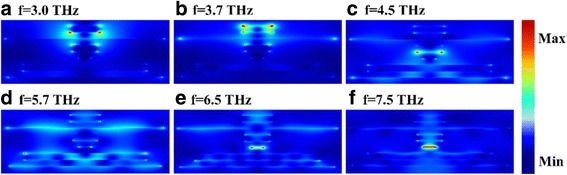
**a**–**f** The distributions of the electric field amplitude (|*E*|) of the proposed absorber at different frequencies

**Fig. 7:**
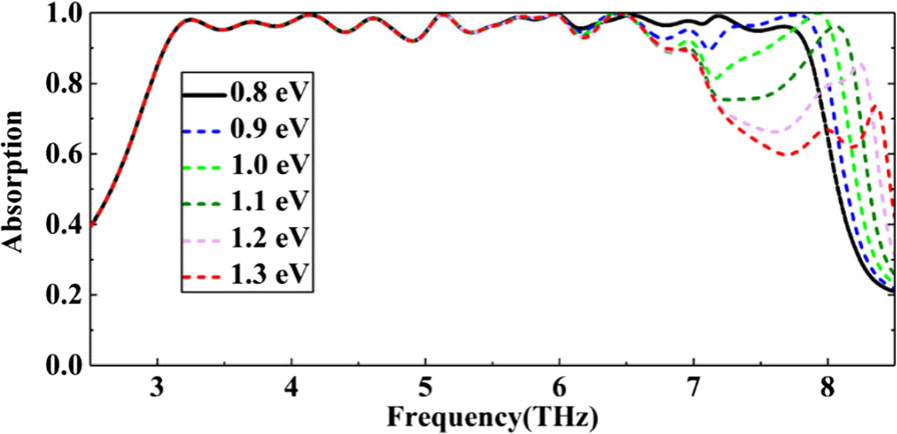
Absorption spectra with different *E*_*f*_ of the fourth layer of graphene and with unchanged *E*_*f*_ of other layers of graphene

As shown in Fig. 4b, the structure has a near-perfect absorption bandwidth of 1.3 Thz with a center frequency of 5.25 Thz. Three resonance peaks at 4.7, 5.2, and 5.7 Thz are obtained corresponding to the absorption amplitude of 99.9, 99.9, and 99.1%, respectively. In order to achieve an ultra-broadband absorption, similar to the three-layer graphene structure, we add more graphene layers and adjust the graphene parameters of each graphene layer to the appropriate values. We assume that the structural parameters are fixed and the production is completed; we can dynamically adjust the Fermi energy of graphene to achieve broadband absorption. Based on the principle of impedance matching and the research experience of three-layer graphene structure, we first assume that the Fermi level of each layer of graphene is 1 eV. As shown in Fig. 5 (a), the absorption of most bands is over 90% except for the band “1” and “2”. Figure 5 (a–e) shows the gradual adjustment process for perfect absorption of the “1” and “2” bands. According to Fig. 6e, f, the absorption of the last band “1” is dominated by the fourth layer (from bottom to top), so we adjust the Fermi energy of this layer individually. As shown in Fig. 7, when the Fermi energy is 0.8 eV, the absorption performance is best. This is because the Fermi energy affects the impedance of graphene, and then affects the input impedance of the whole structure. The larger or smaller Fermi energy of graphene will lead to impedance mismatch. From a to b, we have improved the absorption performance of the “1” band (in the band before “1”, curves a and b are approximately overlapping). Similarly, we find that the energy distribution in the “2” band is mainly concentrated on the 5th, 8th, and 9th layers. We first set the Fermi energy of 8th and 9th layers of graphene to 0.9 and 0.8 eV, respectively. As shown in Fig. 5, from b to c, in addition to dip “3” and “4”, the absorption of the remaining band in “2” is over 90%. Then, according to Fig. 6c, dip “3” is mainly influenced by the 5th layer of graphene, we set the Fermi energy to 0.8 eV. From c to d, the absorption performance at dip “3” has also been improved. However, according to Fig. 6d, dip “4” is affected by all layers of graphene. Therefore, we adjust the Fermi energy of the remaining graphene layer to the appropriate value. From d to e, the near-perfect broadband absorption is achieved. Compared with the three-layer graphene structure shown in Fig. 4, more resonance absorption peaks are obtained, absorption peaks of different frequencies are close to each other and superimposed to form an ultra-broadband absorption above 90% with a bandwidth of 4.8 Thz.

To understand the physical mechanism behind the ultra-broadband near-perfect absorption, we also give a detailed calculation and analysis about the electric field amplitude (|E|) distributions of the proposed structure at different operating frequency. As shown in Fig. 6, the energy of the light field is confined between the different layers of graphene and dielectric, leading to a strong absorption. The characteristics of the electric field distributions are consistent with the absorption spectrum shown in Fig. 2. At a certain frequency, for example, Fig. 6b shows the electric field confinement is mainly due to the strong coupling of graphene and dielectric owing to the excitation of localized surface plasmon (LSP), Fig. 6d shows the graphene surface plasmas play a major role in the electric field confinement. The excitation of localized surface plasmon (LSP) and graphene surface plasmas contribute to the strong absorption together. Figure 6a, b, d and Fig. 6c, e, f show that the strong coupling between graphene and dielectric at a certain frequency may be caused by multi-layer graphene or monolayer graphene, respectively. The stacking of high absorption at different frequencies creates a broadband absorption under the action of all layers of graphene.

In order to better illustrate the stacking effect, for example, according to Fig. 6e, f, the absorption of the last band (about 6.5 Thz later) is mainly dominated by the fourth layer of graphene (from bottom to top). So, we tune the voltage of this layer of graphene. As shown in Fig. 7, with the increase of the Fermi energy of the fourth layer of graphene, the absorption amplitude of the band after about 6.5 Thz gradually increases, but there is almost no change in the band before 6.5 Thz. Similarly, we can also independently adjust a certain band mainly affected by other layers of graphene. All bands which can be adjusted independently for high absorption are superimposed to form a broadband absorption eventually. As with the analysis of Fig. 7, the phenomenon of independent adjustment further illustrates that the stacking effect of all layers of graphene achieves a near-perfect broadband absorption.

**Fig. 8:**
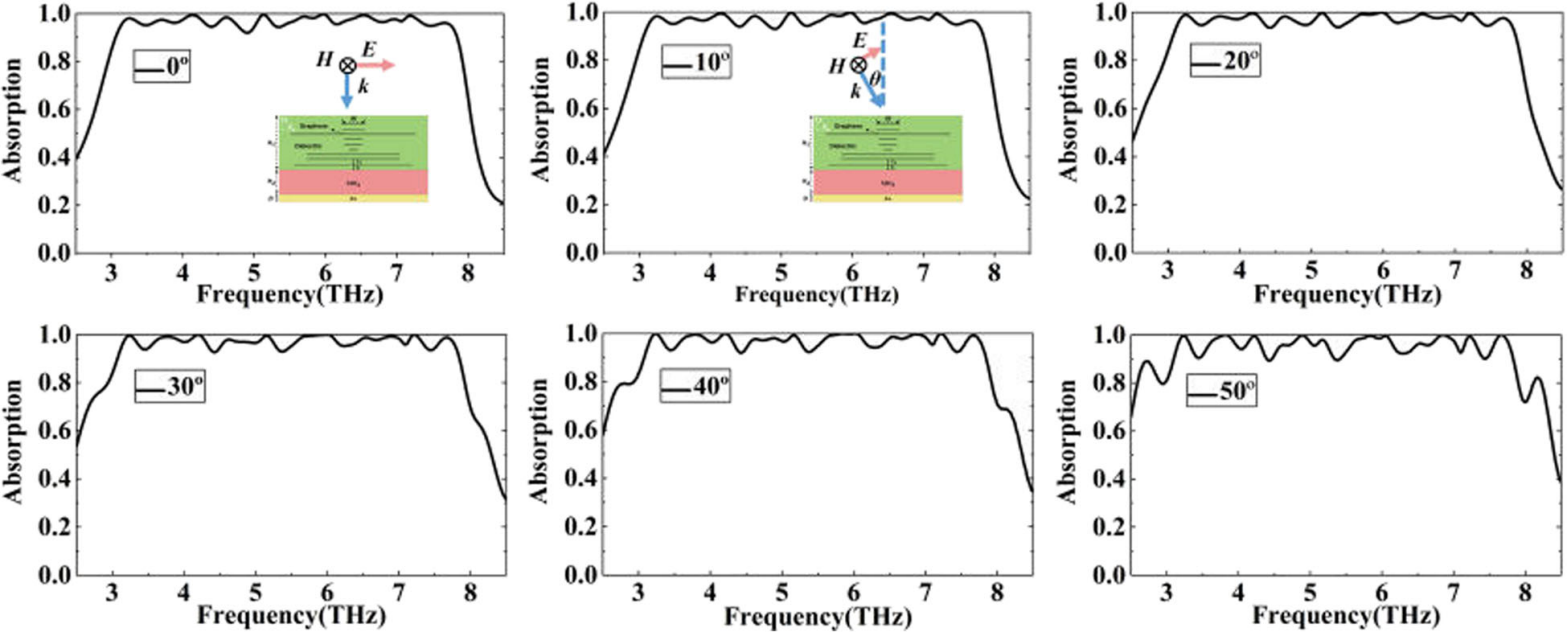
The calculated absorption spectra of the absorber with different incident angles

As discussed above, the strong coupling between graphene and the dielectric plays a major role in broadband absorption. In the practical applications, we hope that the broadband absorption is insensitive to the incident angles. As shown in Fig. 8, we investigate the effect of the incident angles on the absorber. From Fig. 8, we can find that the proposed absorber is insensitive to incident angles. Although the incident angle has changed to 30°, the absorption performance of the structure is almost unaffected. As the incident angle increases to 50°, although the absorption efficiency is reduced, the absorber still maintains a high absorption more than 90% in the whole operating bandwidth. Therefore, the absorber can work well with high absorption efficiency over a large range of incident angle.

**Fig. 9:**
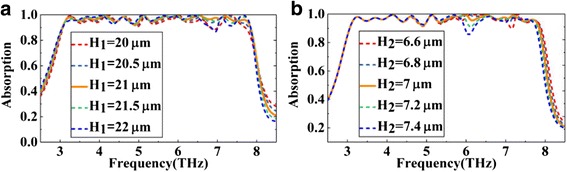
The simulated absorption spectra of the proposed absorber with different thickness of the dielectric layer and with different thickness of the SiO_2_ layer corresponding to **a** and **b**

Finally, considering the difficulties of multi-layer structure in fabrication, we discuss the effect of the relevant structure parameters on the absorber performance. Figure 9a, b show the absorption spectra of the proposed absorber with different thickness of the dielectric layer *H*_1_ and with different thickness of the SiO_2_ layer *H*_2_, respectively. As shown in Fig. 9a, the most suitable height of the dielectric *H*_1_ is 21 μm. On this basis, *H*_1_ increase or decrease 0.5 μm, the performance of the absorber almost has no change. Even if *H*_1_ changes by 1 μm, the absorber still maintains an absorption above 90% in most bands except the band around 7 Thz. As shown in Fig. 9b, compared with *H*_1_, the absorber is more sensitive to the height of the SiO_2_*H*_2_. Even in this case, in addition to the band around 6 and 7.1 Thz, the absorber also maintains a good performance in the most bands. As discussed above, we can find that although the thickness of the dielectric layer and the SiO_2_ layer are changed even at the micron scale, the absorber still maintains a good absorption performance in most wavelengths which will greatly improve the robustness of the absorber in the fabrication.

## Conclusions

In this paper, we propose an ultra-broadband, tunable graphene-based terahertz absorber consisting of multilayers of graphene/dielectric. The proposed absorber can achieve a broadband absorption over 90% with a bandwidth of 4.8 Thz through altering the Fermi energy *E*_*f*_ of different graphene layers. With the *E*_*f*_ = 0 eV, the proposed design will be a near-ideal reflector with reflection more than 90% within the whole operation bandwidth of 3–7.8 Thz. The ultra-broadband absorption is ascribed to the stacking effect of strong resonance absorption at different frequencies excited by localized surface plasmon (LSP) and graphene surface plasmons. In addition, the proposed absorber is insensitive to the incidence angles, and we also find that the thickness of the dielectric layer and the SiO_2_ layer has little effect on the absorption performance, which is more beneficial to practical applications. Moreover, the proposed absorber is simple which do not depend on complex structured graphene and the bandwidth may be widened by adding more graphene layers. This tunable broadband absorber may have great potential applications in photodetectors, imaging, and modulators.

## Abbreviations

FDTD: Finite-difference time-domain
LSP: Localized surface plasmon
